# Poly (ε-Caprolactone)/Cellulose Nanofiber Blend Nanocomposites Containing ZrO2 Nanoparticles: A New Biocompatible Wound Dressing Bandage with Antimicrobial Activity

**DOI:** 10.34172/apb.2020.069

**Published:** 2020-08-09

**Authors:** Sina khanmohammadi, Ramin Karimian, Mojtaba Ghanbari Mehrabani, Bahareh Mehramuz, Khudaverdi Ganbarov, Ladan Ejlali, Asghar Tanomand, Fadhil S. Kamounah, Mohammad Ahangarzadeh Rezaee, Mehdi Yousefi, Elham Sheykhsaran, Hossein Samadi Kafil

**Affiliations:** ^1^Faculty of Chemistry, Department of Organic Chemistry, Azad University of Tabriz, Tabriz, Iran.; ^2^Chemical Injuries Research Center, Systems biology and poisonings institute, Baqiyatallah University of Medical Sciences, Tehran, Iran.; ^3^Drug Applied Research Center, Tabriz University of Medical Sciences, Tabriz, Iran.; ^4^Connective Tissues Diseases Research Center, Tabriz University of Medical Sciences, Tabriz, Iran.; ^5^Department of Microbiology, Baku State University, Baku, Republic of Azerbaijan.; ^6^Department of Basic Sciences, Faculty of Medicine, Maragheh University of Medical Sciences, Maragheh, Iran.; ^7^Department of Chemistry, University of Copenhagen, Universitetsparken 5, DK-2100 Copenhagen, Denmark.; ^8^Infectious and Tropical Diseases Research Center, Tabriz University of Medical Sciences, Tabriz, Iran.; ^9^Stem Cell and Regenerative Medicine Institute, Tabriz University of Medical Sciences, Tabriz, Iran.

**Keywords:** Antimicrobial activity, Cellulose, MTT, Nanocomposites, Polycaprolactone, Solvent exchange, Thermal properties, Zirconium dioxide

## Abstract

***Purpose:*** In the present study, the poly (ε-caprolactone)/cellulose nanofiber containing ZrO_2_ nanoparticles (PCL/CNF/ZrO_2_ ) nanocomposite was synthesized for wound dressing bandage with antimicrobial activity.

***Methods:*** PCL/CNF/ZrO_2_ nanocomposite was synthesized in three different zirconium dioxide amount (0.5, 1, 2%). Also the prepared nanocomposites were characterized by Infrared spectroscopy (FT-IR), X-ray diffraction (XRD), differential scanning calorimetry (DSC), and thermogravimetric analysis (TGA). In addition, the morphology of the samples was observed by scanning electron microscopy (SEM).

***Results:*** Analysis of the XRD spectra showed a preserved structure for PCL semi-crystalline in nanocomposites and an increase in the concentrations of ZrO_2_ nanoparticles, the structure of nanocomposite was amorphous as well. The results of TGA, DTA, DSC showed thermal stability and strength properties for the nanocomposites which were more thermal stable and thermal integrate compared to PCL. The contact angles of the nanocomposites narrowed as the amount of ZrO_2_ in the structure increased. The evaluation of biological activities showed that the PCL/CNF/ZrO_2_ nanocomposite with various concentrations of ZrO_2_ nanoparticles exhibited moderate to good antimicrobial activity against all tested bacterial and fungal strains. Furthermore, cytocompatibility of the scaffolds was assessed by MTT assay and cell viability studies proved the non-toxic nature of the nanocomposites.

***Conclusion:*** The results show that the biodegradability of nanocomposite has advantages that can be used as wound dressing.

## Introduction

Nowadays the production of polymer nanocomposites has dramatically grown.^1,2^ In recent years, the focus of research and scientific studies is to simultaneously exploit the properties of the semiconductor polymers, as well as the properties of metal oxides for the production of new polymer nanocomposites including better physical and chemical properties.^1,3-5^ Using the cellulosic nanofibers, due to their unique features and capabilities, has resulted in the production of high-quality nanocomposites.^6^ The addition of nanofibers to polymers leads to emergence of better physical, chemical and mechanical properties in nanocomposites. Researchers developed the production of different nanocomposites by adding cellulosic nanofibers to different types of polymers, which could be mentioned: poly(l-lactide-co-glycolide) PLGA, poly (l-lactic acid)–poly (d-lactic acid) PLLA–PDLA, poly (dl-lactide-co-glycolide)–poly (ε-caprolactone) PLGA–PCL, poly (ε-caprolactone)-poly (l-lacticacid) PCL–PLLA, poly (l-lactic acid-co-ε-caprolactone) PLCL, poly (l-lactic acid-ε-caprolactone), P(LLA–CL).^7-12^

It is important to note that the polymer properties improve with the addition of cellulosic nanofibers. Regarding the PCL, due to its hydrophobicity, its physical and chemical properties and its biodegradability are better in the lake of water, while in the moist medium such as biofilters or biosensors, it is difficult to use these compounds.^13,14^ In contrast, cellulosic nanofibers have a high water absorption capability.^6^ Due to its unique chemical and physical properties such as high strength, chemical stability, high resistance to corrosion and microbial and chemical agents, zirconium dioxide has always been a matter of interest to researchers.^15,16^ Zirconium dioxide is one of the most important mediator metal oxides used in many fields such as oxygen sensors, optical coatings, fuel cells, electrochemical devices, catalysts and dielectrics.^17^ With the rapid development of nanotechnology, polymer nanocomposites containing nanoparticles were rapidly expanding.^18,19^ In recent years, many researchers have come up with new polymer nanocomposites using metal oxide nanoparticles that create unique chemical properties.^20,21^ Cellulose as one of the most important natural polymers is a biocompatible and enduring material on industrial scale. It has been used for many years as wood and herbal fibers as an energy source, construction materials and apparel.^22,23^

Polycaprolactone (PCL) is a semi-crystalline polyester. It acts extremely slow due to its semi-crystalline nature and high hydrophobic property.^24^ As a result, PCL is combined with other polymers in the form of composite to both control and increase the rate of degradation and improvement of cellular adhesion properties. PCL is prepared by open-loop polymerization of ε-caprolactone using a catalyst. Recently, a wide range of catalysts for caprolactone loop polymerization has been used.^25^ The aim of this research was to produce the desired nanocomposite. The solvent exchange method was carried out in order to preparation of PCL/CNF/ZrO_2_ nanocomposite for the first time and in this paper, we investigated the antibacterial properties of the nanocomposites using Zirconium dioxide nanoparticles.

## Materials and Methods

### Materials

The materials used in this study were as follows: Polycaprolactone (Purchased from Sigma-Aldrich, Missouri, United States), cellulose nanofiber (CNF) (Obtained from Nano Novin Polymer Co, Tehran, Iran). Zirconium dioxide nanoparticles (50-100 nm) (Purchased from US Research Nanomaterials, Houston, United States). Mueller Hinton Agar (MHA) (Purchased from Liofilchem, Province of Teramo, Italy). *Staphylococcus aureus* (ATCC 29213), *Escherichia coli* (ATCC 25922), and *Candida albicans* (NRRL Y-477) strains (Obtained from Microbiology Department of Drug Applied Research Centre, Tabriz, Iran (DARC)). Fibroblast cell line L929 (NCBI C161) used in the cytotoxicity studying (Purchased from Cell Bank, Pasteur Institute of Tehran, Iran). All other materials were purchased from Merck.

### Characterization

#### Fourier transform infrared spectroscopy (FT-IR)

FT-IR absorption spectra were carried out using a single beam Fourier transform-infrared spectrometer (FTIR 84000s Bruker, Germany). The FT-IR nanocomposite produced by using KBr tablets in the range of 1400-4000 cm^-1^.

#### X-ray diffraction

The X-ray diffraction spectra (XRD),( D8 advanced Bruker, Germany), were acquired that equipped using Cu-Ka radiation (λ = 1.540562 Ǻ, the tube operated at 40kV, Bragg’s angle (2θ) in the range (4-80°).

#### Scanning electron microscopy

The morphology of the films was characterized by scanning electron microscope using (LEO1455 VPSEMjena, Germany), operating at 200 kV accelerating voltage. The surface of the samples was coated with a thin layer of gold by the vacuum evaporation technique to minimize sample charging effects due to the electron beam. Scanning electron microscopy (SEM), (SEM LED 1430VP, USA) examined the morphology of nanofibers. All samples were sputtered with Carbon before assessment.

#### Differential scanning calorimetry

Thermal behavior of the PCL/ZrO_2_ composites was studied using a differential scanning calorimetry (DSC) (Mettler Toledo- DSC 821, Columbus, Ohio, United States), differential scanning calorimeter in a nitrogen atmosphere. In the DSC method, heating the samples and their scans were recorded. Heating range of samples was selected 0 to 400°C and rate 23°C/min and held at that temperature for 10 minutes in order to remove any previous thermal history. During reheating, the melting parameters were recorded from the scans.

#### Thermal gravimetric analysis

TGA experiments were conducted using a (TGA/DTA STA PT-1000, Princeton Junction New Jersey, USA) equipment at different heating speeds including 25-800°C under a heating rate of 10°C/min in nitrogen and oxygen atmosphere (60 mL/min). Samples about 10 mg were placed in alumina crucibles and tested with a thermal ramp over a temperature range of 25 to 600°C. The weight loss of the samples during heating was automatically recorded and plotted as a function of temperature.

### Preparation of nanocomposite

The solvent casting method was performed to manufacture PCL. One gram of PCL granules was dissolved in 25 mL of acetone at 45°C using the ultrasonic bath (IKA RCT basic, Germany). The obtained mixture is then placed in a glass plate under the hood to completely dry the film at room temperature (25°C). Due to the dissolution of PCL in organic solvents, CNF (2.5 w/v%) and PCL are mixed together. In order to produce PCL/CNF (90:10), the mixture was disintegrated for 45 minutes on magnetic stirrers (FALC Instruments LBS2 15 Lt Treviglio (BG)

**Figure 1:**
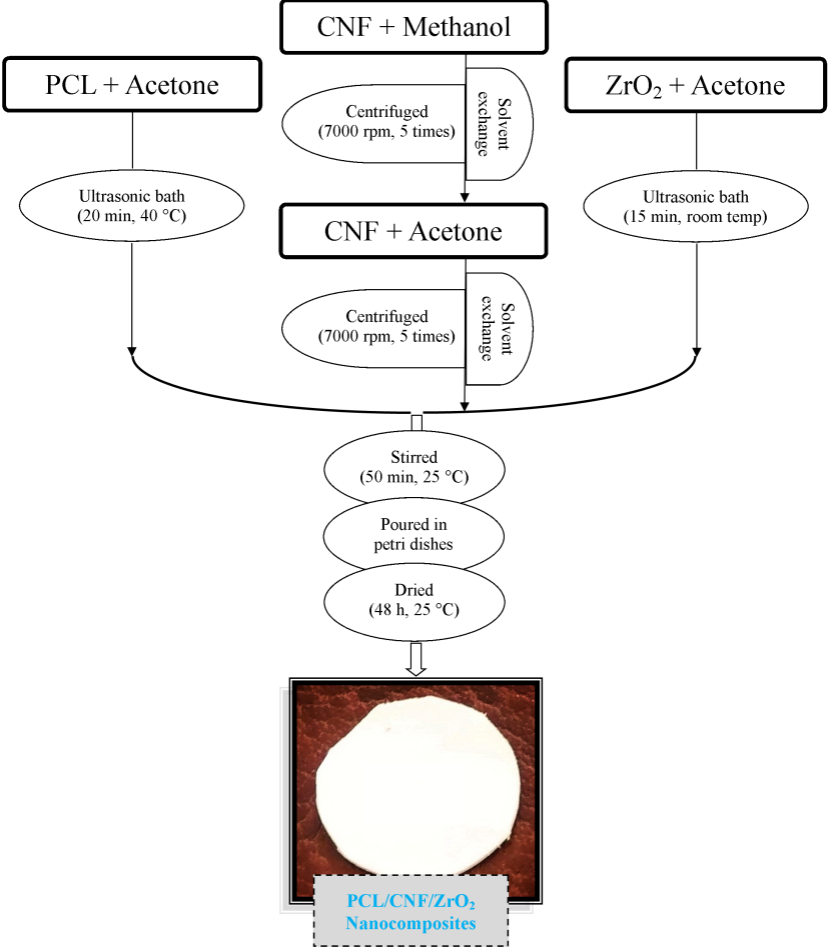
Flowchart of PCL/CNF/ZrO_2_ nanocomposites preparation.

ITALIA,) and finally, the mixture poured onto a glass plate. Therefore, we used the solvent exchange method for CNFs and PCL/CNF/ZrO_2_ nanocomposite, which is able to gradually replaced water with a polarized solvent such as Methanol, after that, organic solvents, acetone (25 mL) is instead Methanol by centrifugation (Hettich EBA 88, Kirchlengern, Germany). For this purpose, the mixture centrifuged by 5862 g for 5-6 times. Then the glass plate was coated by PCL/CNF/ZrO_2_ nanocomposite films and placed under the hood for 48 hours at room temperature to be dried (Figure 1).

### Water solubility 

The solubility test was performed according to the method reported by Ojagh et al.^26^ Briefly, dried pieces of prepared nanocomposites were cut into small equal discs including 10 mm diameter. The composite specimens were weighted (Mi), immersed in 20 mL of distilled water and kept at room temperature for 24 hours. After this period, the samples were taken from the petri dishes, gently rinsing all pieces with distilled water, dried at 50°C for 24 hours and weighed again (Mf). All experiments were performed in triplicate, and the percentage of water solubility was calculated according to the following equation:

Water – solubility (%) = [(Mi – Mf)/Mi] × 100

### Water contact angle 

The measurement of wetting level related to a solid surface by a liquid, contact angle is used. In this quantitative method, the surface area is a key factor, the higher surface is associated with lower contact angles. A water droplet was poured on the surface of solid samples and the contact angle was recorded using a digital camera. In order to increase its reliability, 5 measurements were performed for each scaffold type.

### Evaluation of antibacterial and antifungal activity

The antimicrobial activities of the PCL, PCL/CNF, and PCL/CNF/ZrO_2_ nanocomposites were tested according to the Clinical & Laboratory Standards Institute (CLSI) Kirby-Bauer Disc diffusion method. *S. aureus* and *E. coli* were taken as model gram-positive and gram-negative bacteria. *Candida albicans* was used as a fungal strain. Muller-Hinton agar and Muller-Hinton agar plus 1% glucose containing 2% glucose were employed as a culture medium for bacterial and fungal strains, respectively.^27^ All organisms suspensions were adjusted to 0.5 McFarland standards (including a final bacterial concentration of 1.5×10^8^ CFU.mL^-1^). The Mueller-Hinton agar media were inoculated with suspension using a sterile cotton swab. The samples were cut into the disc shape with a 6 mm diameter by a punch machine. The discs were sterilized with 75% ethanol for 30 min and placed in ultraviolet (UV) radiation for second 30 minutes. A pellet of each nanocomposite is transferred on cultured plates and incubated at 37ºC for 24 hours. Sterile Whatman filter paper discs with and without gentamicin (10 μL, for bacterial) and fluconazole (25 μL, for fungal) was used as a positive and negative control.^28^ After incubation, the inhibition zone diameters were measured in millimeters, using a caliper ruler. For all the strains the disc diffusion tests were performed in triplicate and the results were expressed as mean ± SD.^29,30^

### Evaluation of cytotoxicity (MTT assay)

One of the best indirect methods available to determine the cell proliferation is the test for dimethyl tiazole diphenyl tetrazolium bromide (MTT, Purchased from Sigma-Aldrich, Missouri, USA) based on the change of the tetrazolium yellow powder to the violet insoluble crystalline Formazan.^27,31^ This phenomenon occurs only in living cells and using the enzyme present in their mitochondria which is called succinate dehydrogenase enzyme. Formazan crystals can be solubilized using an organic solvent such as isopropanol, and the optical density (OD) obtained by the ELISA reader. The OD is proportional to the formazan concentration, and also for metabolic activity of the living cells. In this study, for cell proliferation, first 1×10^4^ cells in a volume of 150 μL culture were placed on each sterile specimen contained within each well of a 96-well cell culture plate. After 3 days, the media was removed from the cells as much as possible and 150 μL of the MTT solution at 0.5 mg/mL was poured into each well and placed in an incubator for 4 hours. After 4 hours, the solution was removed from cells and the isopropanol was added to dissolving the obtained purple crystals. To better dissolve MTT sediment, the plate was placed on a shaker for 15 minutes. in the next step, the amount of 100 μL of purple solution per well was transferred to the 96-well plate. Then, the concentration of dissolved material in isopropanol was calculated using ELISA reader device (STAT FAX 2100, USA) at a wavelength of 570 nm. These wells have higher cells and higher OD compared to wells including low cells. Therefore, the equation (1) can be used to determine the value of the wells with high cells and compare with the control sample. It should be noted that each sample has 3 replications.^27,32^

$$\begin{array}{l} Toxicity(\%)=(1-mean\;of\;OD\;sample/mean\;of\;OD\;control)\times 100\\ Viability(\%)=100-Toxicity(\%) \end{array}$$

### Statistical analysis

Statistical analysis was performed using GraphPad Prism software (v.6.07). Data were expressed as the mean ± SD. *P* value at less than 0.05 was considered to statistically significant.

## Results and Discussion

### FT-IR characterization

**Figure 2:**
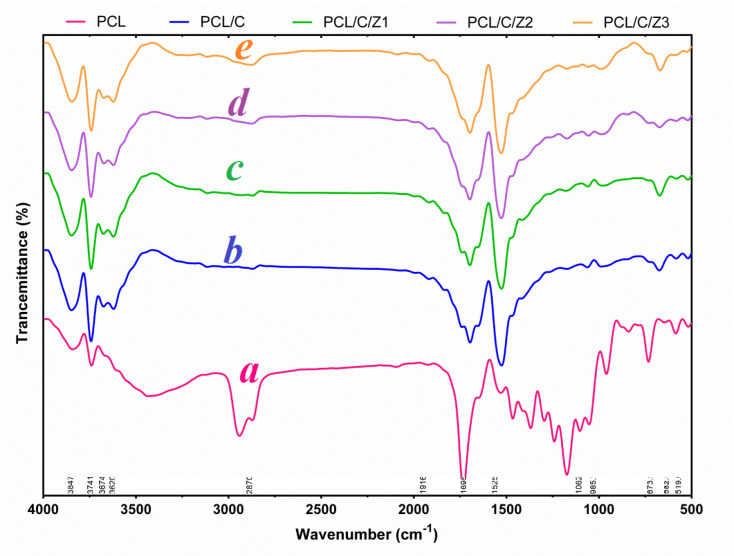
FT-IR spectra of nanocomposites nanocomposites (a) pure polycaprolactone, (b) PCL/C is polycaprolactone/cellulose nanofiber, (c) PCL/C/ Z1 is Polycaprolactone/cellulose nanofiber/ZrO_2_ NPs 0.5%, (d) PCL/C/ Z2 is Polycaprolactone/cellulose nanofiber/ZrO_2_ NPs 1%, (e) PCL/C/Z3 is Polycaprolactone/cellulose nanofiber/ZrO_2_ NPs 2%.

The infrared spectra of pure PCL, PCL/CNF, and PCL/CNF/ZrO_2_ are shown in Figure 2. The courier appeared to be about 1000 cm^-1^ in relation to the symmetric and asymmetric traction CH_2_, the carbon tetragonal stretch and the symmetric stretch (C-O-C) of 1726 and 1243 cm^-1^, respectively. Regarding the spectra obtained from PCL/CNF samples, the symmetric and asymmetric tensile peaks of about 1300 cm^-1^ are removed and indicate the formation of the composition and the presence of peaks at about 1650 cm^-1^ indicates the presence of carbonylene stretch in the structure nanocomposite. Such a peak exists in PCL/CNF nanocomposites. The absorbance peaks in the 3300–3400 cm^−1^ and 1634–1640 cm^−1^ are attributed to the stretching vibrations of the OH groups of cellulose and the elongation of adsorbed water molecules, respectively.^33-36^

### XRD analysis

**Figure 3:**
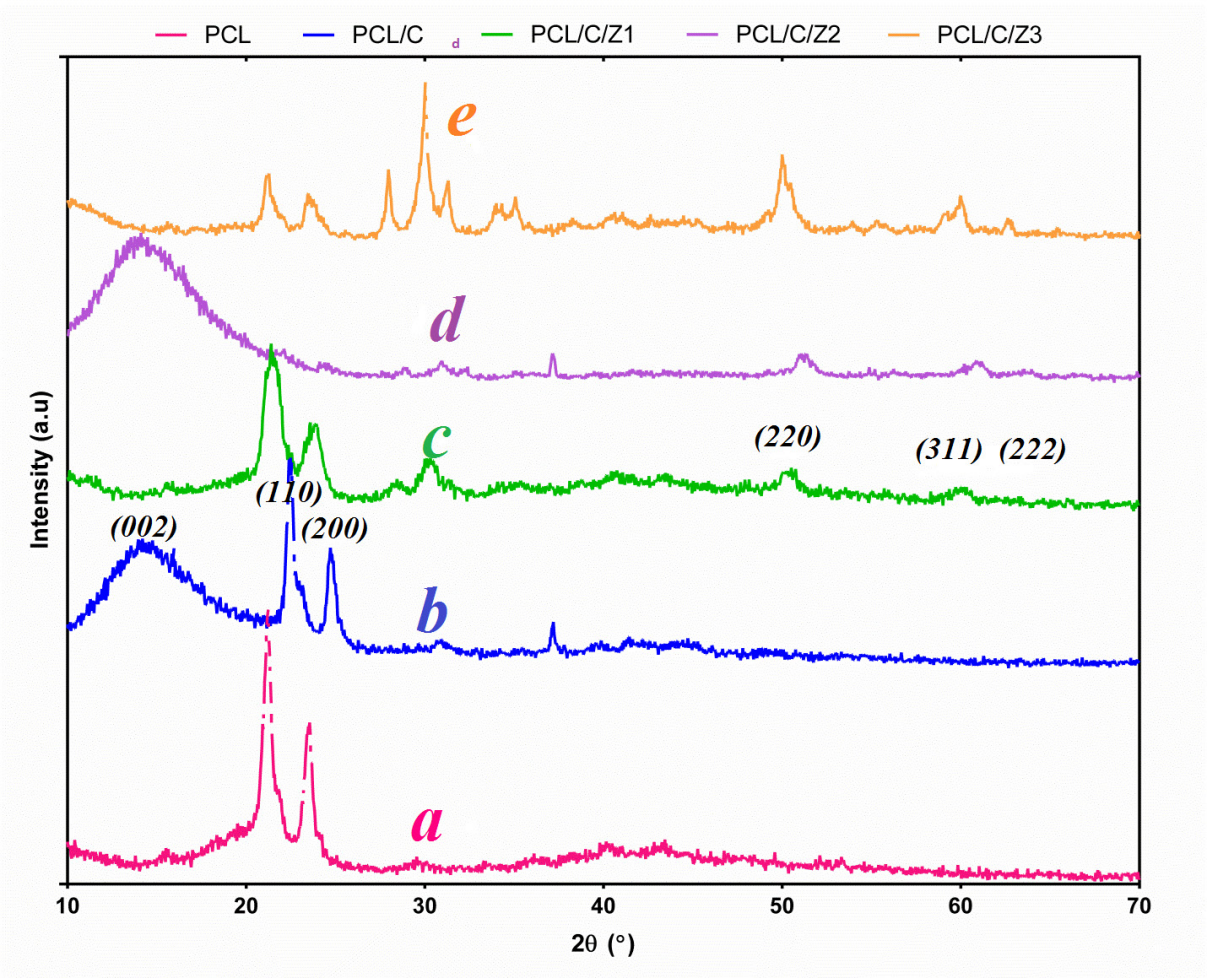
XRD patterns of nanocomposites (a), polycaprolactone/cellulose nanofiber (b), and nanocomposite with 0.5% (c), 1% (d) and 2% (e) ZrO_2_ NPs.

XRD analysis have been performed on neat PCL, PCL/CNF and nanocomposite containing a different percentage of ZrO_2_ NPs (Figure 3). In the spectrum obtained for the XRD, two peaks in the spectrum are quite evident, with a strong differential peak at 23 degrees and a relatively weak peak at 21 degrees, indicating the PCL semi-crystalline state. The observed distinct pixel in these nanocomposites, indicate the preservation of semi-crystalline state in PCL. When the intensity of the differential peaks is reduced, it indicates the proper percentage of the nanoparticles filler.^37^ The ZrO_2_ nanoparticles are surrounded by a thin layer of PCL filled with cellulosic fiber filler cations. In this case, there are distinct peaks between PCL/CNF nanocomposite and PCL/CNF/ZrO_2_ nanocomposite, which changed the PCL state.^33^ An increasement in the concentrations of ZrO_2_ nanoparticles, the intensity and the area under the peak at 2θ=23° and 26° decreased is observed. It implies to a decreasement in degree of crystallization and an enhancement of amorphous structure of nanocomposite, which in turn increases the homogeny of the PCL/ZrO_2_ films. This behavior demonstrates that blending between the ZrO_2_ and PCL takes place in the amorphous region.

### Morphological studies

**Figure 4:**
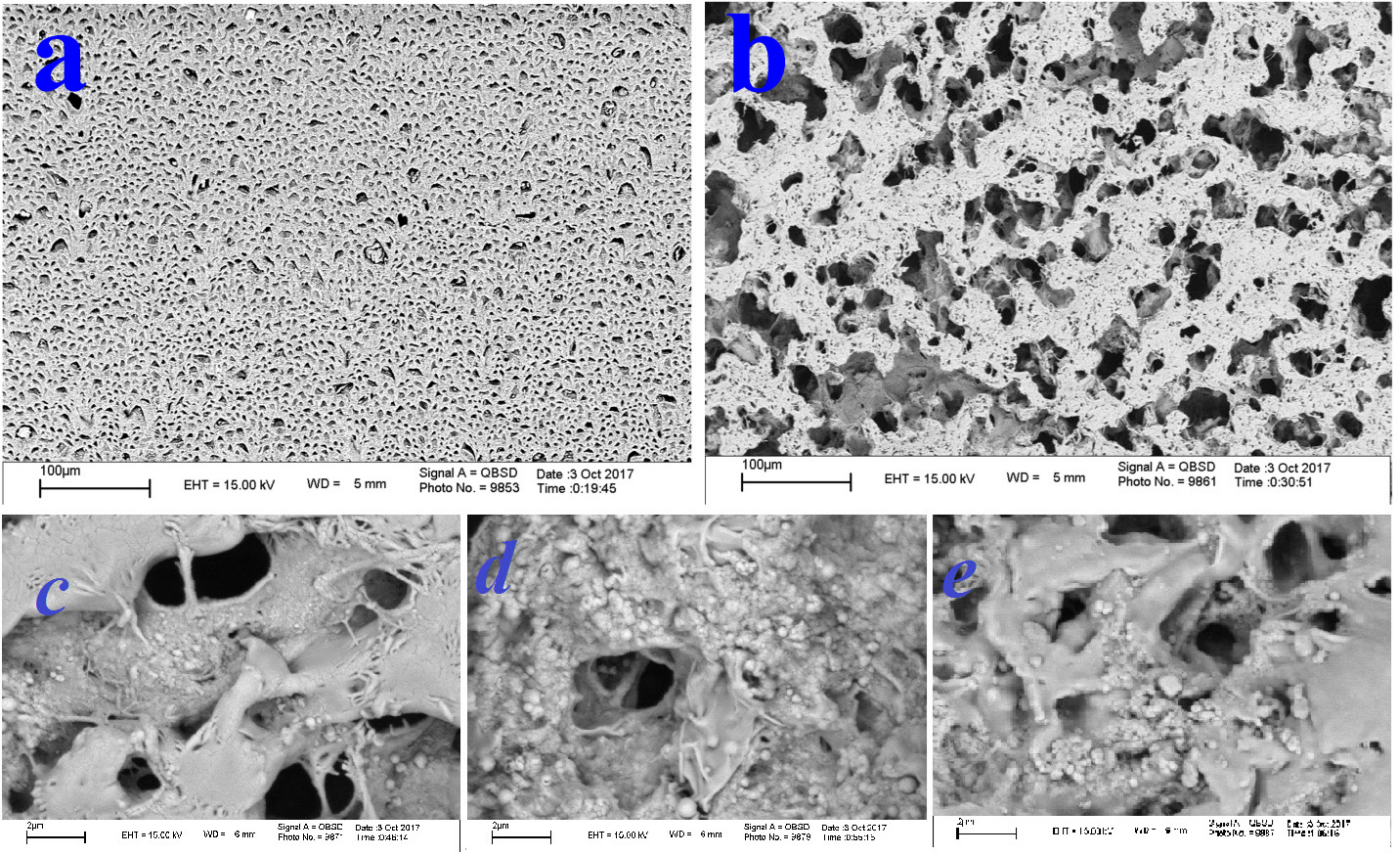
The SEM micrographs of pure polycaprolactone (a), polycaprolactone/cellulose nanofiber (b), and nanocomposite with 0.5% (c), 1% (d) and 2% ZrO_2_ NPs.

The SEM images of the PCL/CNF/ZrO_2_ nanocomposite are shown in Figure 4. We observed that the number of cavities in the PCL/CNF/ZrO_2_ nanocomposite structure is lower than the PCL structure and a coherent structure was created by adding cellulosic nanoparticles and nanoparticles to PCL/CNF/ZrO_2_. SEM images revealed that the PCL nanofibers add mechanical stability have occurred in the nanocomposites.

### Thermal properties

**Figure 5:**
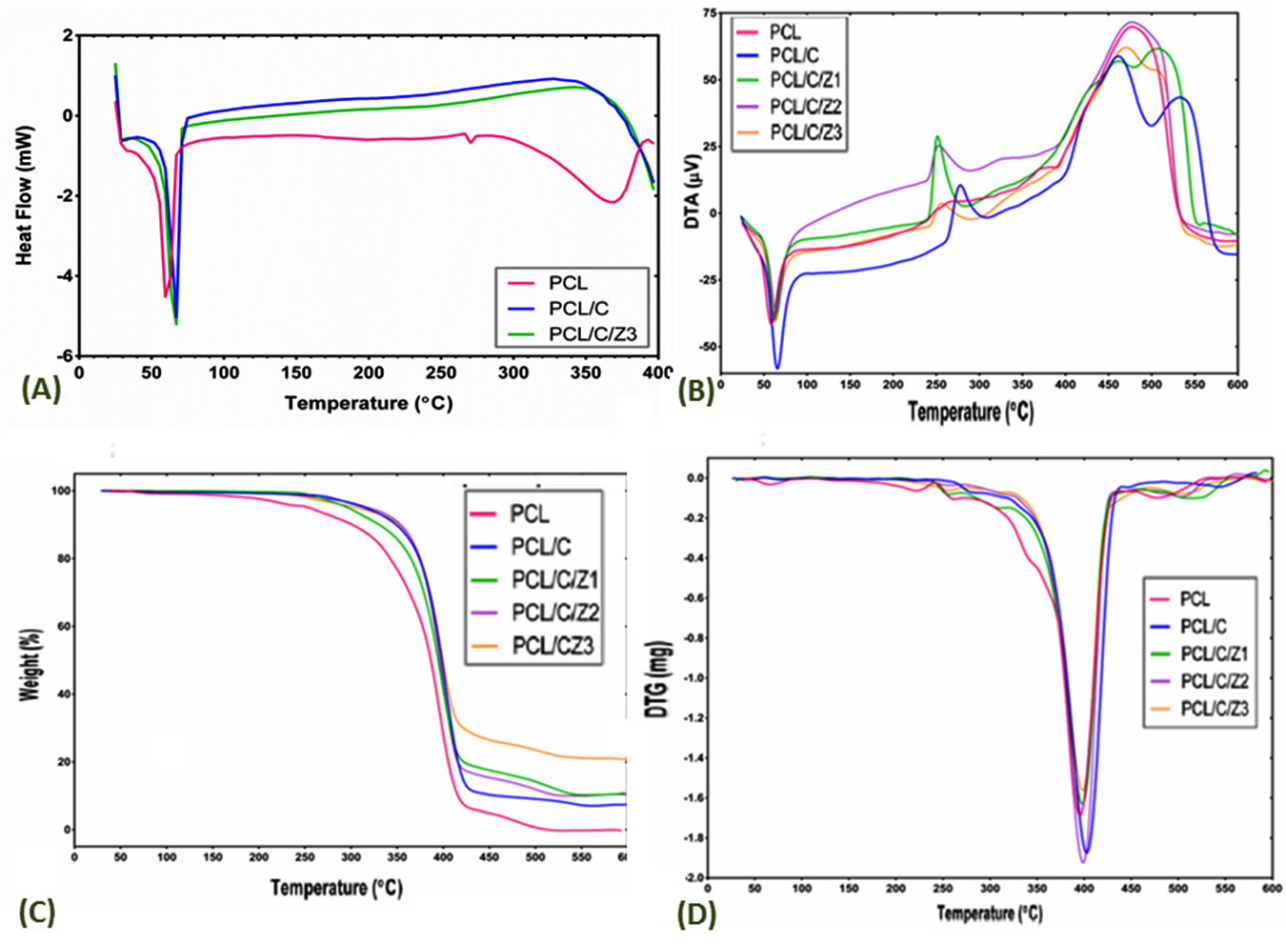
(A): DSC curves of samples. PCL is pure polycaprolactone, PCL/C is PCL/CNF, PCL/C/Z1 is PCL/CNF/ ZrO_2_ NPs 0.5%, PCL/C/Z2 is PCL/CNF/ ZrO_2_ NPs 1%, and PCL/C/Z3 is PCL/CNF / ZrO_2_ NPs 2%.(B):DTA, (C): TGA, and (D): DTG thermograms of nanocomposites. PCL is pure polycaprolactone, PCL/C is PCL/CNF, PCL/C/Z1 is PCL/CNF/ ZrO_2_ NPs 0.5%, PCL/C/Z2 is PCL/CNF/ ZrO_2_ NPs 1%, PCL/C/Z3 is PCL/CNF/ ZrO_2_ NPs 2%

For studying the thermal behavior of the nanocomposites, DSC was used (Figure 5). Table 1 shows a summary of the thermal parameters obtained from the DSC thermograms for PCL, PCL/CNF, and PCL/CNF/ZrO_2_ (1%) nanocomposite.

The crystallinity percentage (χ_c_) of PCL and its composites were calculated using the heat of composites fusion to the heat of fusion of the completely crystalline polymer ratio as an external standard:

χc=ΔHmωΔHom

where ΔH_m_ and ΔH^°^_m_ are the enthalpy of fusion per gram of the samples (recalculated for the PCL mass), the enthalpy of fusion per gram of 100% crystalline PCL respectively and ω is the weight fraction of polymeric matrix in the composite. In this paper, ΔH^°^_m_ was equaled 142.0 J/g.^38,39^ In Table 1, melting temperature (𝑇_𝑚_), enthalpy of fusion (Δ𝐻_𝑚_), crystallization temperature (𝑇_𝑐_), degree of crystallinity (χ_c_), decomposition temperature (𝑇_𝑑_), are defined for all the samples.

**Table 1:** Statistical results of the thermal properties

	**PCL**	**PCL/CNF**	**PCL/CNF/ZrO**_2_
T_m_ (°C)	60.37	66.70	65.67
T_d_ (°C)	367.69	373.01	377.83
ΔH_m_	235.35	238.52	264.98
χ_c_	1.11±0.09	1.11±0.1	1.11±0.1

According to the results of DSC, it can be seen that by adding ZrO_2_ nanoparticles to nanocomposite films, the nanocomposite degradation temperature has increased and the produced film has a higher coherence. Also, due to amorphous structure of nanocomposite and used polymer (cellulose) in its structure, the change in the crystallization of compounds is not considered.

### Thermogravimetric analysis

Figure 5B shows the thermal stability of all the investigated samples. It is observed that the addition of cellulosic nanofibers and ZrO_2_ nanoparticles to the PCL structure results in the integrity and structural strength of the nanocomposite, which is seen from the thermal disruption of the nanocomposite produced at higher temperatures. In pure PCL at several temperatures, mainly at low temperatures, the structure is destroyed by heat. Thermogravimetric analysis (TGA), as an analytical technique, is used to differentiate the volatile components of weight change which occurs in the heated sample. TGA thermograms are shown in Figure 5 and it is raveled that weight loss is as a function of temperature for pure PCL and its nanocomposites as the sample are heated at 10 °C/min in the temperature range from room temperature to 600°C.^40^

In Figure 5C, the TGA curve indicates that PCL/CNF/ZrO_2_ nanocomposites are stable until 190°C and any decomposition occurred in the above 197°C. The TGA curves of the PCL/CNF/ZrO_2_ nanocomposites indicated three step degradation processes, the first degradation of nanocomposite has occurred in 197°C and its destruction continues in 229, 464°C. PCL/CNF/ZrO_2_ nanocomposite has completely decomposed in 593°C. According to earlier studies, the thermal stability generally increases in pure polymer films due to the presence of micro/nanoparticles.^41,42^

### Solubility test

The percentage of water solubility of PCL, PCL/CNF and nanocomposite containing the different percentage of ZrO_2_ was measured. As expected, PCL was insoluble in water and did not display any solubility. The result also showed that the addition of CNF and ZrO_2_ nano particles has not a significant effect on PCL solubility. This finding may be attributed to the strong interactions between ZrO_2_, CNF, and PCL in the polymer structure. Thus, the prepared nanocomposites presented 0% of solubility in distilled water.

### Surface hydrophobicity

**Figure 6:**
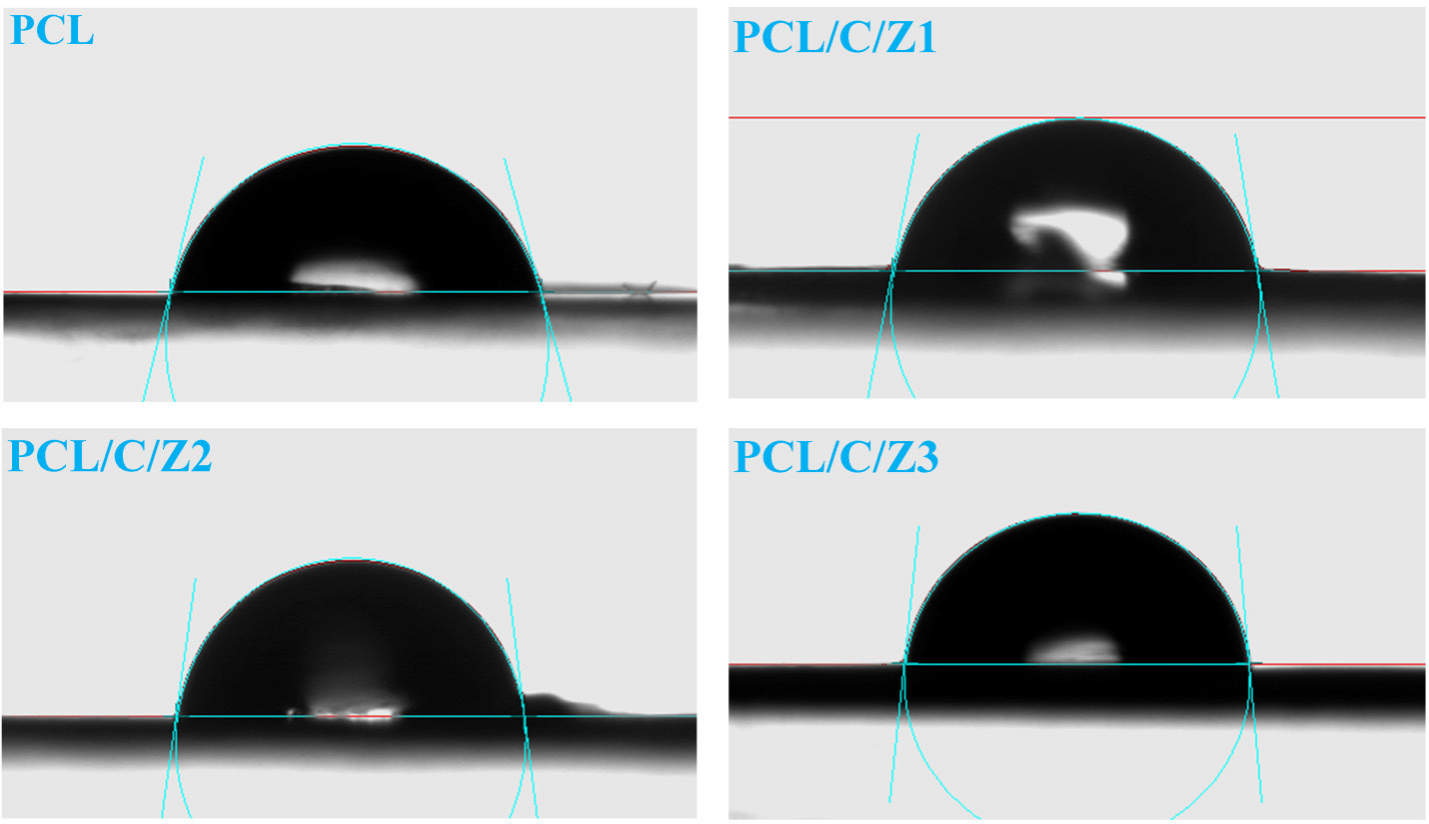
The static water contact angle of the produced nanocomposites. PCL is pure polycaprolactone, PCL/C is PCL/CNF, PCL/C/Z1 is PCL/CNF/ ZrO_2_ NPs 0.5%, PCL/C/Z2 is PCL/CNF/ ZrO_2_ NPs 1%, PCL/C/Z3 is PCL/CNF/ ZrO_2_ NPs 2%.

The contact angle made by the drops of the water on the film surfaces is shown in Figure 6. It can be observed that PCL surface had a higher contact angle (75.5°), it is be respected to have a hydrophobic nature.^43^ Furthermore, CNF is more hydrophilic than PCL, as result, the addition of CNF to PCL, the surface wettability of the composite increased and so, the contact angle significantly decreased.^44^ Lastly, the hydrophobicity of the scaffolds was significantly increased by the addition of ZrO_2_ and hydrophobicity of PCL/CNF/ZrO_2_ nanocomposites increased when the amount of ZrO_2_ in the composites increased from 0.5 to 2. Table 2 indicates the incremental trend of hydrophobicity nanocomposites.

**Table 2:** Contact angle measured by the drops of water on surfaces

**Sample**	***θ***
PCL	75.55 ± 1.06
PCL/C/Z1	79.80 ± 1.13
PCL/C/Z2	82.45 ± 0.49
PCL/C/Z3	84.4 ± 0.70

### Antibacterial and antifungal activity of nanocomposites

The results of the antimicrobial evaluation are presented in Table 3. As the results were shown, the pure PCL and PCL/CNF have no zone of inhibition against all strains. It can also be seen that as the nZrO_2_ concentration increased, the zones of inhibition are also increased. The PCL/CNF with higher concentrations of ZrO_2_ nanoparticles exhibited good to moderate activity against both bacterial and fungal microorganisms with an inhibition zone between 6.5 and 9 mm. PCL/CNF/Z2 nanocomposite also inhibited the growth of both *S. aureus* and*C. albicans* , but with less effectivity. PCL/CNF/Z1 exhibited no obvious antimicrobial activities. These data indicate that the antibacterial and antifungal activity was due to the presence of nZrO_2_ in the composites and this nanoparticle exhibits fair antibacterial action against *E. coli* . Overall, it can be concluded that the PCL/CNF/ZrO_2_ is a promising antimicrobial nanocomposite.

**Table 3:** Disc diffusion test results for developed samples against gram positive (*S. aureus* ), gram negative (*E. coli* ) and fungi (*C. albicans* ) pathogens

**Samples**	**Zone of inhibition (mm)**		
	***S. aureus***	***E. coli***	***C. albicans***
PCL	-	-	-
PCL-C	-	-	-
PCL-C-Z1	-	-	-
PCL-C-Z2	7.53 ± 0.15	-	6.90 ± 0.17
PCL-C-Z3	8.76 ± 0.07	6.35 ± 0.22	7.80 ± 0.12
Gentamicin/fluconazole	21.00	18.00	23.00

### Cytotoxicity studies

**Figure 7:**
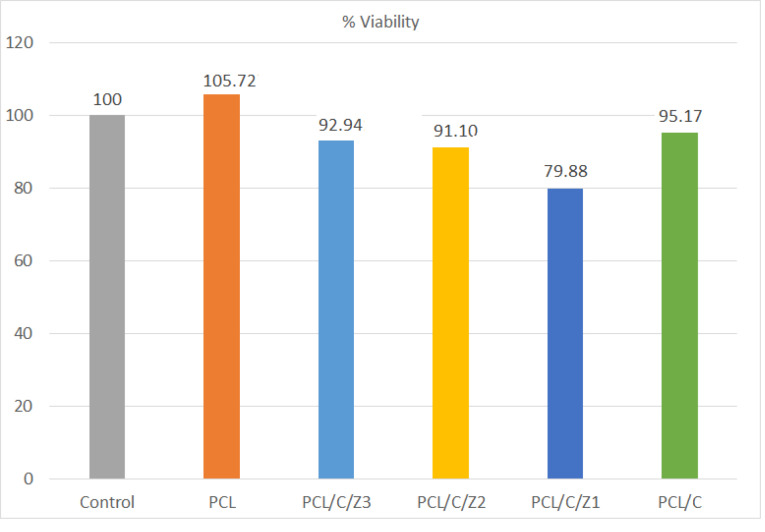
Cell viability evaluation of developed samples using L929 (NCBI C161) cells.

In the current study, the MTT test was used to evaluate the potential cytotoxicity of the prepared nanocomposites. MTT assay results confirmed the biocompatibility of nanocomposites. It seems that in the PCL/CNF/ZrO_2_ nanocomposite the percentage of live cells was not significantly affected after exposure of the cells to all CNF samples at concentrations compared to the positive control group (Figure 7).

## Conclusion

The PCL/CNF/ZrO_2_ nanocomposite in part with a biodegradable PCL polymer and cellulosic nanofibre enhanced with ZrO_2_ nanoparticles has acquired antimicrobial property. The high density of ZrO_2_ nanoparticles and cellulosic nanofibers causes uniformity in the polymer structure of the PCL, which is clearly visible through SEM images. The XRD results indicate that semi-crystalline PCL mode is maintained in the structure of the prepared nanocomposites. It can be observed an increasing in the concentrations of ZrO_2_ nanoparticles, the intensity and a significant decreasement in the area under the peak as well. Therefore, it implies to a decrease in the degree of crystallization and amorphous structure of nanocomposite enhancement, which in turn increases the homogeny of the PCL/ZrO_2_ films. According to earlier studies, the thermal stability generally increases in pure polymer films due to the presence of micro/nanoparticles. The hydrophobicity of the scaffolds was significantly increased as a result of adding ZrO_2_. The hydrophobicity of PCL/CNF/ZrO_2_ nanocomposites was growing also when the amount of ZrO_2_ in the composites increased from 0.5 to 2%. The thermal degradation curve of the nanocomposite shows that the nanocomposite has a higher thermal strength and coherence compared to the PLC. In vitro results of antibacterial and antifungal properties showed that PCL/CNF containing a high percentage of ZrO_2_ nanoparticles were more effective in inhibiting Gram-positive bacteria and fungi growth. The cytotoxicity studies indicated that prepared nanocomposites have no significant cytotoxic effects on L929 (NCBI C161) cells as observed in the results related to MTT assay.

## Ethical Issues

Not applicable.

## Conflict of Interest

None to declare.

## Acknowledgments

This study was supported by Drug Applied Research Center, Tabriz University of Medical Sciences with Reference number 61703 and was approved in local ethic committee with reference number IR.TBZMED.VCR.REC.1397.412. We would like to thank all staff of DARC and Microbiology laboratory of Drug Applied Research Center.
